# The anxious wait: assessing the impact of patient accessible EHRs for breast cancer patients

**DOI:** 10.1186/1472-6947-10-46

**Published:** 2010-09-01

**Authors:** David Wiljer, Kevin J Leonard, Sara Urowitz, Emma Apatu, Christine Massey, Naa Kwarley Quartey, Pamela Catton

**Affiliations:** 1 Department of Radiation Oncology, University of Toronto, Toronto, ON, Canada; 2 Princess Margaret Hospital/University Health Network, Toronto, ON, Canada; 3 Department of Health Policy, Management and Evaluation, University of Toronto, Toronto, ON, Canada; 4 Centre for Global eHealth Innovation, University Health Network, Toronto, ON, Canada; 5 Department of Psychiatry, University of Toronto, Toronto, ON, Canada; 6 Rollins School of Public Health, Emory University, Atlanta, GA, USA

## Abstract

**Background:**

Personal health records (PHRs) provide patients with access to personal health information (PHI) and targeted education. The use of PHRs has the potential to improve a wide range of outcomes, including empowering patients to be more active participants in their care. There are a number of widespread barriers to adoption, including privacy and security considerations. In addition, there are clinical concerns that patients could become anxious or distressed when accessing complex medical information. This study assesses the implementation of a PHR, and its impact on anxiety levels and perceptions of self-efficacy in a sample of breast cancer patients.

**Methods:**

A quasi-experimental pre-test/post-test design was used to collect data from participants to evaluate the use of the PHR. Study participants completed background and pre-assessment questionnaires and were then registered into the portal. By entering an activation key, participants were then able to review their lab results and diagnostic imaging reports. After six weeks, participants completed post-assessment questionnaires and usability heuristics. All data were collected using an online survey tool. Data were cleaned and analyzed using SAS v9.1.

**Results:**

A total of 311 breast cancer patients completed demographic and pre-assessment questionnaires, 250 registered to use the online intervention, and 125 participants completed all required study elements. Matching the pre- and post-anxiety scores demonstrated a decrease in mean anxiety scores (-2.2, p = 0.03); the chemotherapy sub-group had a statistically insignificant mean increase (1.8, p = .14). There was no mean change in self-efficacy scores.

**Conclusions:**

Participants generally found the portal easy to use; however, the perceived value of improved participation was not detected in the self-efficacy scores. Having access to personal health information did not increase anxiety levels. While these results suggest that the use of this PHR may be of benefit for informing patients, further research is required to investigate the impact on the patients experiences, their participation in their care, their relationships with the health care team, and their health outcomes.

## Background

The development of personal health records (PHRs) has wide ranging implications for personal health. Perhaps the greatest opportunity for impact is with patients facing a chronic or life threatening illness. These individuals require information to manage their own health and health care [1-3]. Studies have shown that patients have expressed an interest in having access to their PHRs [4]. PHRs can generally be defined as Internet-accessible applications that allow patients or guardians to create, review, annotate or maintain a record of any aspect of their health condition, including medication, allergies, vaccinations and visit history [5,6]. Depending on their design and implementation, PHRs may capture both objective and subjective health information. Research done by Tang et al., 2006 [2], showed that the information captured in PHRs has the potential to transform the patient-physician relationship. By providing patients with accurate information about the status of their current health, PHRs can facilitate effective communication with members of their health care team, and may help patients to become active members in the management of their illness [2].

PHRs have the potential to provide health information that is not only more tailored to individuals, but also more credible than general information on the Internet [2]. Patients with chronic illnesses will be able to keep track of their diseases and their associated symptoms and treatments. PHRs provide an ongoing connection between patients and physicians [7-10]. PHRs may also result in lower chronic disease management costs; lower medication costs and lower wellness program costs, although these areas need to be evaluated further [11-13]. Previous studies have shown that PHRs are convenient [14,15], and that they have the potential to improve patient adherence to their care plans [5], and increase patient empowerment by encouraging patients to participate in the management of their health [9,16].

There are many barriers to widespread adoption of PHRs. Many of these barriers are associated with an institution's readiness for the adoption of PHRs. Four key areas have been identified that must be addressed to improve institutional readiness: 1) access to the health record, 2) privacy and security, 3) provision of necessary education to use the PHR effectively, and 4) organizational change [17]. The feasibility of addressing issues related to providing patient access and privacy and security has been already demonstrated [18]. The area of education is still not well understood. Further, within the area of organizational change, the impact of having access to a PHR on the patient experience has not been investigated, especially with respect to psychosocial issues such as anxiety, distress and clinician workflow.

Anxiety arising from dealing with health issues is often a part of the patient experience. This is especially true for cancer patients who may experience anxiety as a result of their diagnosis and the treatment of their disease [19]. Stressors can include receiving chemotherapy, watching other patients receive treatments, waiting to see their health care provider, and waiting for results [20]. The anxiety associated with waiting for results is particularly concerning, given that cancer patients typically undergo a range of tests and procedures throughout their cancer trajectory, and spend a considerable amount of time waiting to receive results [21]. PHRs present an opportunity to address this type of anxiety by enabling patients to access their test results as they become available, without having to wait for a clinic visit. Although this can reduce the anxiety associated with waiting, there is the potential of anxiety or distress that might result from accessing troubling results outside of a standard medical consultation. When accessing sensitive information online, patients do not necessarily have immediate access to their health care team, complex results can be misinterpreted or misunderstood and patients may not have timely access to the appropriate resources and support [21].

In an effort to provide timely access to personal health information (PHI) and engage patients as partners in their own care, the Breast Cancer Survivorship Program (BCSP) at Princess Margaret Hospital and the Shared Information Management Services (SIMS) at University Health Network, in Ontario, Canada, developed a PHR called InfoWell. InfoWell offers patients real-time access to elements of their institutional health record, such as laboratory results, diagnostic imaging, and pathology reports. It also provides educational resources and tools to assist in the management of care, such as appointment schedules and medication lists [22,23].

Initially developed and piloted for patients with chronic kidney disease, diabetes or breast cancer, InfoWell is currently being used to investigate issues related to the adoption of PHRs. There are two objectives of the present analysis; 1) to determine the feasibility of using the InfoWell platform and 2) to explore how access to a secure patient portal impacts on two clinical outcomes, anxiety levels and self-perceptions of self-efficacy.

## Methods

### Design

A quasi-experimental pre/post test design was used to evaluate usability and measure changes in levels of anxiety and self-efficacy. This study was approved by the University Health Network (UHN), Research Ethics Board and written informed consent was obtained.

### Procedure

Convenience sampling was used to recruit 250 breast cancer patients from the BCSP to use InfoWell. These patients have diverse and often complex needs that span from the time of diagnosis, and persist through treatment and often for many years later. Late effects of breast cancer can include, but are not limited to, psychosocial distress, upper body lymphedema, infertility, hot flashes caused by estrogen deprivation, fatigue, cardiovascular disease, and cognitive impairment [24]. Eligible participants were breast cancer patients at any stage. Participants had to have access to a computer, be able to complete all instruments in English, and have no cognitive impairments. Clinical staff and research assistants approached potential participants during regular appointments and provided them with a brief introduction to the portal and the study. After written consent was obtained, the participants completed self-administered questionnaires (background, demographics and a set of pre-assessment measures). Participants were then registered to use InfoWell for a six week period. At the end of the intervention period, a link to an online post-assessment survey and usability heuristic was sent to participants; two follow-up reminders were sent at one-week intervals.

### Online Intervention

InfoWell is a secure, PHR that enables users to access elements of their personal health information [22]. Users can access lab results, including complete blood count (CBC), chemistry results and liver function tests. They can also view diagnostic reports including CT, MRI, ultrasound and pathology. All of the results are linked directly to the electronic medical record and available to the patient when they are entered into the system. Personal health information in InfoWell is both generated by the patient and from the institutional electronic health record accessed through InfoWell. Within InfoWell, users can organize and record information about their health care and view their appointment schedules. InfoWell also supports a patient profile, medication lists and treatment history section.

In addition, InfoWell provides information that spans three content domains: general health, experiential and personal health. General Health information focuses on disease specific information, different treatment types, members of the health care team, and links to additional resources such as emergency information and printed materials. Experiential information includes access to a virtual librarian, as well as support groups that are available in face-to-face and online formats.

InfoWell is designed to be a secure environment. In order to view lab results and diagnostic reports, the patients must enter a password and their medical record number (MRN) along with a unique 16-character activation key. Patients are able to access results and reports as far back as 6 months prior to their account registration date. All results and reports remain accessible for a 6 month period. Laboratory results could take up to 3 days to be posted; imaging reports up to 3 weeks. Finally, participants were provided with access to technical, clinical, educational and psychosocial support through a telephone or email-based call centre, the research triage centre (RTC) [23]. The role of the RTC was to determine the type of support required, and direct the participant to the appropriate resources. Any technical issues were handled directly by the RTC, whereas other support needs were addressed by clinicians (typically a social worker) [23].

### Study Instruments

The demographic questionnaire captured age, education, first language spoken, computer and Internet usage, and perceptions of online access to information. The pre-assessment questionnaire investigated issues related to current treatment and access to health related information, including current treatment received, wait times for test and imaging results, usefulness of online access to information, and levels of self-management, in addition to standardized and validated measures for anxiety and self-efficacy. The final post-assessment questionnaire investigated access to test and imaging reports online, perceptions of online access to information and levels of self-management, their satisfaction and experience with the intervention, as well as repeated measures for anxiety and self-efficacy.

Both the anxiety and self-efficacy measures are validated instruments. The State-Trait Anxiety Inventory (STAI) is a self-report anxiety measure. This study focused only on the state measure of anxiety. Use of the STAI allowed us to focus on state anxiety, which would presumably be impacted if access to the PHR resulted in increased situational related anxiety. The state-anxiety is a measure of 20 individual questions, which are all scored between 1 and 4. The anxiety score is the weighted sum of the scores to the 20 individual questions and is calculated only if at least 18 questions are answered. The maximum possible score is 80 and the minimum possible score is 20. Internal consistency is high (r > .90) [25]. The STAI is widely used and norms are reported for normal adults, with a mean score of 35.20 for working adult females with a standard deviation of 10.61 and an alpha coefficient of .93 [26].

The Stanford Self-Efficacy for Managing Chronic Disease is a 6-Item Scale based on a larger self-efficacy scale first developed for the Chronic Disease Self-Management study of multiple chronic illnesses [27]. The scale covers several domains that are common across many chronic diseases, including symptom control, role functioning, emotional functioning and communication with physicians. Each item is measured on a 10-point Likert scale ranging from not at all confident (1) to totally confident (10). When previously tested on 605 subjects with chronic disease the mean score was 5.17 SD 2.22 with an internal consistency reliability of .91.

The usability heuristic employed was a modified Perceived Health Web Site Usability Questionnaire (PHWSUQ). This 16-item instrument has been tested in previous studies and explores the users' experience with online applications, including questions about how easy it is to use, whether technical problems were encountered, whether the information was useful, etc. Out of all the usability questionnaires available in the literature, none were more suitable to assess older adult online users' perceived usability of health Web sites than the PHWSUQ [28].

### Data Analysis

All analyses were performed using SAS v9.1 (SAS Institute Inc., Cary, NC, USA). Responses to the questionnaires were collected using the online survey software, QuestionPro.com [29]. All reported *p-values *are 2-sided and the statistical significant level was set at 0.05 for all tests. Patient summaries, frequencies and proportions were tabulated using *proc freq*. Means, skewness and other descriptives were calculated using *proc means*.

The cases with missing values were not included in the calculation of the *p-values*. The representativeness of the respondents who completed both the pre- and post-test to the full population of respondents was measured using a number of variables from the background and demographic questionnaire. For binary variables (e.g., sex, language) Fisher's exact test was used; for ordered categorical variables, the row mean scores test was used. For education, the 'other' category was treated as missing.

Self-efficacy and anxiety scores from patients who were matched between pre- and post-assessments and who had completed enough of the individual scale or index items to allow for calculation of both the pre- and post-scores were used to perform tests of association between scores and timing of scores (pre- versus post-assessment) and other variables. *Proc univariate *was used to perform signed rank tests for changes in the mean anxiety and self-efficacy scores between the pre- and post-assessments.

Evidence was sought to determine if some of the background variables were related to the amount of change in anxiety from the pre to the post measures. Univariate tests were conducted to determine if the post anxiety scores were related to background variables while controlling for pre-anxiety in a linear regression model. This method was used as it is generally a more powerful method for testing whether the change is related to other variables [30]. All potential predictors were dichotomized: English is first language (yes versus no), age (<= 60 versus >= 61), education (completed high school or less versus some college or more), hours per week on the Internet (<= 10 versus >10), opinion of the reliability of Internet health information (somewhat reliable or less versus mostly or very reliable) and importance of privacy in influencing the choice of whether or not to access health information online (important or very important versus somewhat or less important).

Multivariate analysis was conducted to determine if there was evidence suggesting that post-anxiety scores were related to background variables when controlling for pre-anxiety. The outcome was the post-anxiety score and the model was run in a stepwise fashion, with the pre-anxiety score remaining in the models, removing one predictor variable at a time starting with the variable with the highest p-value.

Responses to open-ended questions included as part of the questionnaires were collated and coded thematically.

## Results

**Figure 1:**
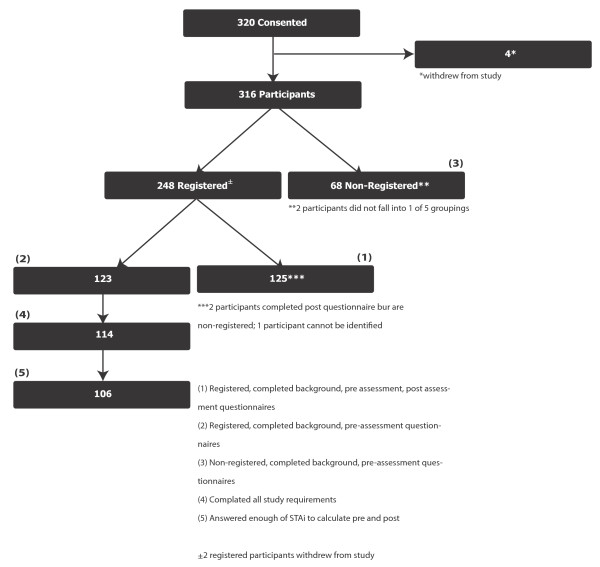
Study Flow

In order to reach the target accrual of 250 participants registered on InfoWell, 320 breast cancer patients were recruited for this study. Three hundred and eleven of the 320 participants who provided initial consent (Figure 1), completed demographic and pre-assessment questionnaires. One hundred twenty-five participants (125) completed all required elements of the study.

Of the participants who completed all required data elements, 114 pre- and post-assessment responses were matched. When the 114 were compared to the remaining 197 patients who completed the background questionnaire, the matched patients access the Internet more often than the remaining patients (p = 0.010), have been using the Internet longer (p = 0.032) and tend to have shorter waiting times for imaging results from PMH (p = 0.020). In addition, matched patients were more likely to have had surgery (p = 0.002) and to be using biological treatments (p = 0.008). There is not strong evidence that the two groups differ on any of the other variables tested, including age, education and English as a second language status.

### Background Questionnaire

Almost all participants (99.7%, 303/304) in this study were female, 75.9% (236/311) were less than 60 years of age, and were well educated (Table 1). Many, 77.8% (242/311), reported English as their first language and 93.8% (289/308) were 'very comfortable' receiving health information in English. 84.6% (263/311) of participants 'often' or 'always' access the Internet; 76% (235/308) have used the Internet for more than 5 years and 39.1% (120/307) used the Internet for more than 10 hours per week. Only 46.4% (140/302) perceived health information on the Internet to be 'mostly' or 'very' reliable.

**Table 1:** Participant Demographics

		N (%)
Gender (n = 304)	Female	303 (99.7%)
	Male	1 (0.3%)

Age (n = 311)	<60 years	236 (75.9%)
	>60 years	75 (24.1%)

Education (n = 310)	Elementary School	3 (0.9%)
	High School	35 (11.3%)
	College/University	177 (57.1%)
	Post-Graduate	83 (26.8%)
	Other	12 (3.9%)

### Pre-Intervention Assessment

64.4% of participants (188/292) reported that they were currently on active treatment; these patients were currently receiving some form of treatment from the hospital for their cancer (e.g., surgery, radiation, chemotherapy etc.) (Table 2). 59.6% of participants (168/282) reported typically waiting less than 10 days to receive the results of lab tests and 60.1% (166/276) for imaging results/reports. 76.9% (240/312) reported that they receive test results from their doctor at their next visit, 18.6% (58/312) reported 'over the phone through their doctor's secretary' and 12.2% (38/312) reported 'over the phone through their nurse'.

**Table 2:** Active Treatment

		N (%)
Are you currently on active treatment? (n = 292)	Yes	188 (64.4%)
	No	104 (35.6%)

Type of Treatment	Surgery (n = 278)	32 (11.5%)
	Radiation Therapy (n = 278)	22 (7.9%)
	Chemotherapy (n = 275)	53 (19.1%)
	Biological (n = 278)	49 (17.6%)
	Other (n = 278)	34 (12.2%)

Almost all respondents, 98.0% (300/306), reported that having access to their personal electronic health record would help them manage their care. 99.7% (310/311) stated that online access to their laboratory test results would be helpful and 95.2% (296/311) indicated online back-up support should be available. 99.4% (310/312) thought that online access to their imaging test reports would be helpful and 96.1% (298/310) indicated online back-up support should be available.

The mean anxiety score was 39.2, the median was 38.0, (standard deviation = 11.5) and skewness of 0.55 (n = 298). The mean self-efficacy score was 7.2, the median was 7.5, (standard deviation = 1.9) and skewness of -0.9 (n = 304).

### Post-Intervention Assessment

64.8% (83/128) of participants indicated that they had viewed their lab test results online and 66.7% (86/129) had viewed their imaging test reports. 95.0% (114/120) indicated that learning to use InfoWell was easy, 95.8% (115/120) indicated that InfoWell would help them improve their knowledge about their health and 95.8% (114/119) indicated that they would recommend InfoWell to other cancer survivors.

Almost all, 97.6%, of the respondents (124/127) perceived that having access to their personal electronic health record would help them manage their care better. 98.4% of respondents (127/129) reported that online access to their laboratory test results would be helpful; 94.5% (121/128) indicated online back-up support should be available. 98.4% (125/127) stated that online access to their imaging test reports would be helpful and 96% (120/125) indicated online back-up support should be available.

The mean anxiety score was 37.6, the median was 38.0, (standard deviation = 10.1) and skewness of 0.60 (n = 123). The mean self-efficacy score was 7.2 with median of 7.4, (standard deviation = 1.6) and skewness of -1.0 (n = 124).

Through open-ended feedback opportunities in the post-assessment questionnaire, participants reported having difficulty using some of the InfoWell applications such as entering information about their medications, accessing information about their care team, searching through the library applications and entering information about their treatment. Some users had difficulty logging into the site and found that navigating through InfoWell was not always intuitive. Some participants reported being disappointed that many of their lab results were not available through the portal and would like retroactive results to be posted, as well as having an online dictionary to define unfamiliar medical terms. At the same time, several respondents stated that receiving information about their test and imaging reports was helpful in the understanding of their disease and also prepared them for the visit with their physicians.

### Testing for Changes from Pre-Assessment to Post-Assessment

114 participants completed all of the study requirements and could have their responses to the background, pre and post questionnaires matched. 106 of those 114 participants answered enough of the anxiety questions that both their pre- and post-anxiety scores could be calculated.

For participants with matched pre- and post-assessments, the mean pre-assessment anxiety score was 40.1 and post-assessment score was 37.9, with a mean change of -2.2. Participants were less anxious when filling out their post-assessment questionnaires (n = 106, signed rank statistic = -633.5, *p *= .03) (Table 3). For chemotherapy patients with matched pre-/post-anxiety scores, the mean pre-assessment anxiety score was 36.2 and post- was 38.0, with a mean change of 1.8. This result was not statistically significant (n = 19, signed rank statistic = 34, *p *= .14) (Table 3). Amongst participants whose pre- and post-assessments for self-efficacy were matched, the mean of both the pre- and post-assessments was 7.1; there was no mean change in self-efficacy scores. According to the signed rank test, there was not enough evidence to suggest a difference in the mean levels of self-efficacy between the pre- and post-assessments (n = 110, signed rank statistic = -109, *p *= .73) (Table 3).

**Table 3:** Changes in Anxiety and Self-Efficacy

Patients	Variable	Mean Pre Score	Mean Post Score	Mean Change	Signed Rank Stat.	p	n
All matched pts	Anxiety	40.1	37.9	-2.2	-633.5	0.03	106
Matched chemo pts	Anxiety	36.2	38.0	1.8	34.0	0.14	19
All matched pts	Self-Efficacy	7.1	7.1	0.0	-109	0.73	110

When controlling for the pre-anxiety score in a linear regression model, there was some evidence to suggest that considering privacy to be important or very important is associated with post-anxiety scores that were lower on average by 5.3, although the result was not quite significant at 0.05. There was not sufficient evidence to suggest that any other background variable was related to post-anxiety scores when controlling for pre-anxiety (Table 4). Results of a multivariate linear regression suggests that there was no evidence that any of the background variables were related to the post-test anxiety scores when controlling for pre-test anxiety scores (Table 5).

**Table 4:** Univariate Linear Regression Model Predicting for Correlation between select Demographics and Change in Anxiety

Variable	Adjusted Parameter Estimate	Standard Error	t Value	Pr > |t|
English is first language	0.17994	2.26765	0.08	0.9369
Age	-2.36136	2.20290	-1.07	0.2863
Education	1.51698	3.24230	0.47	0.6409
Internet Hours	0.20967	1.84882	0.11	0.9099
Reliability of Info	-0.72866	1.80734	-0.40	0.6877
Importance of Privacy	-5.28321	2.80586	-1.88	0.0625

**Table 5:** Multivariate Linear Regression Model Predicting for Correlations between Background Demographics and Change in Anxiety

Step	Variable Removed	Partial R-Square	Model R-Square	C(p)	F Value	Pr > F
1	Education	0.0002	0.2586	6.0299	0.03	0.8632
2	Internet Hours	0.0002	0.2584	4.0595	0.03	0.8629
3	Reliability of Info	0.0005	0.2579	2.1210	0.06	0.8029
4	English is first language	0.0012	0.2567	0.2715	0.16	0.6945
5	Age	0.0123	0.2444	-0.1522	1.64	0.2036
6	Importance of Privacy	0.0254	0.2191	1.0980	3.36	0.0699

## Discussion

This study suggests that providing patients with access to their personal health information through a PHR may have a positive impact on their experience. Other studies have demonstrated that providing patients with access to personal health information online is feasible [10,14,15,31]. Studies have also suggested that providing patients with access to this information online has the potential to reduce the time that patients wait for results, to empower patients to better manage their care, to improve decision making and to improve communication between health care providers and patients [2,32-34]. At the same time, concerns have been raised about the potential harm that this access could have on patients and their families. The concerns range from patients accessing information without understanding the clinical significance of the results, to misunderstanding the information, and receiving bad news without the appropriate clinical or educational support, causing anxiety and undue distress for patients and providers alike [17,21,23].

The results from this study suggest that providing access to personal health information may not impact negatively on patient anxiety. Although not all participants completed both the pre- and post-questionnaires, data gathered from the pre-questionnaires are useful in providing insight into the first objective of determining the feasibility of using this type of online portal. The participants who completed both sets of study instruments tended to use the Internet more and have been using it longer. Our findings support the idea that computer literacy is a common enabler for use of PHR. It is therefore possible that a lack of familiarity with the Internet resulted in some participants not using InfoWell. It is not, however, possible, with the data collected to correlate InfoWell usage with the completion of study measures. Those who completed all study measures also had surgery and were using biologic therapies which may have provided them with additional reason to access InfoWell and complete the post-intervention measures.

Our participants want access to reliable health information provided by their hospital. With respect to their PHI, the majority of study participants strongly preferred the option of accessing health information from a hospital-based system, in comparison to community, industry or government options. In addition, privacy, security, convenience and timeliness were all perceived to be important factors influencing a participant's likelihood of using InfoWell; this finding is not surprising given that other studies have cited the importance of privacy, security and convenience [15,32].

Participants found InfoWell easy to use and helpful; 65% of participants reported viewing lab results and 68% reported viewing diagnostic imaging reports. At first glance, this number may appear lower than expected, especially since 98% of participants (pre- and post-intervention) reported that having access to their results would help them better manage their care. However, the relatively lower usage rate is most likely attributable to the fact that 35% of participants were not on active treatment and only 19% of those on treatment were receiving chemotherapy during the time when they were first recruited onto InfoWell. Therefore, many participants may have had limited lab results and imaging reports to view during the study period, since these are more prevalently used during periods of active treatment.

Providing patients access to their results requires information and support [23]. Participants did not change their opinion through the course of the intervention about the importance of educational resources; 65% of both pre- and post-surveys reported a perceived value in having resources for test results and 62% for diagnostic imaging reports. For both lab test results and imaging reports, the majority of participants in the pre- and post-assessments reported that online back-up support should be available for those accessing the results and/or reports [23].

As expected in a cancer population, anxiety levels for both the pre- and post-tests were above the average range for a normal population [35]. For the general population of women, we would expect a value of 35.20 [25]. Of those whose pre- and post-assessments were matched, the mean pre-score was 39.1 and the post- was 37.9, a statistically significant mean decrease of 2.2. Although there was a 1.8 increase in anxiety levels for chemotherapy patients, it was statistically insignificant. There was a great deal of concern among some clinical staff that there would be an increase in anxiety because of the release of results. This may be related to the fear of patients receiving bad news when viewing imaging or testing results, or a preference for providing these results in person, during a consultation with a physician. This outcome was not detected in this study. We cannot directly correlate changes in anxiety scores to this intervention, since it has been demonstrated that a wide range of factors impact on anxiety scores for cancer patients [36] and distress decreases for breast cancer patients over time [37]. Previous studies investigating anxiety related to the wait for PSA results in prostate cancer patients concluded that rapid results did not alleviate the anxiety related to receiving results, but patients did prefer it [38]. However, the findings from this study do suggest further investigation is required to better understand the impact of the intervention on breast cancer patients.

We sought to determine if access to InfoWell could increase patient self-efficacy and patient participation in their care. No change in self-efficacy was detected. This lack of results could pertain to the instrument used. In addition, it could suggest that the information on InfoWell was not sufficient to give participants a sense of confidence in managing their care. Although some information was tailored based on the results, it may not have been sufficient; the tailored content was limited to the test results and was not provided for the diagnostic imaging reports.

Participants who did not perceive information on the Internet to be reliable were less likely to have improved self-efficacy scores after using InfoWell, whereas those participants who perceive online information to be reliable where more likely to have improved self-efficacy scores after using the portal. In the background questionnaire, 86% of participants 'agreed' or 'strongly agreed' that having reliable information approved from the hospital would make them feel more able to make decisions about their treatment and disease. This suggests that the source of the information may not necessarily be the primary or only factor impacting on the sense of confidence that an individual has in the content they receive or in their ability to act on that information. There are many factors that impact on the perception of health information. Studies have shown that many patients still prefer to obtain information from physicians [39-41] but that they may go to the Internet first [39]. Education, socio-economic status, and cultural background may all impact on the way information on the Internet is accessed perceived and used [42-44]. Information on the Internet is of varying quality [45] and readability [46]. All of these factors may impact on the relationship between the perception of information and self-efficacy. Additional research is required to understand more completely the relationship between the reliability of information and a patient's sense of self-efficacy.

### Limitations

The study had several limitations associated with the implementation of a new technology. There were several technical difficulties in the beginning of the study related to passwords, activation keys and temporary Internet pages. In terms of overall usage, only 78% of the participants who provided initial consent actually registered into the portal; this may be in part due to technical issues, despite the fact that technical support was provided [23]. This may also suggest that patients previously received the information from their health care providers, and as such, did not have a need to go online to retrieve the results and associated information for themselves. There were limitations on what results were available and how far back results were reported. The educational content available was not comprehensive, as this was a pilot test rather than a full implementation and certain features such as appointment schedules became available part way through the study period, which may have skewed the results.

Furthermore, almost half of the registered participants did not complete all of their instruments and therefore became ineligible for the matched comparisons. For this reason, it was not possible to draw adequate conclusions about sub-groups in the study such as those on active chemotherapy. In addition, participants of the study were at different points in the continuum of care. While 64% were on active treatment, many participants were several years post-treatment. Skewed samples may have been due to the fact that participants in this study were highly educated, and also appear, from their experience with the Internet, to be technology adopters and therefore not representative of our patient population as a whole. Another limitation was that there was a difference in assessment collection, the post-assessment surveys were only sent online. Thus there was a lack of follow-through among those who did not use the site after registration. Finally, the results of the study are limited to patients who used InfoWell with no comparison to a control group. As such, it is difficult to determine whether impacts on patient satisfaction or self-efficacy are due to the global effect of the information system, or the PHR. Given that the study design was hypothesis generating, no firm conclusions about anxiety and self-efficacy can be made without randomized control trials being conducted.

## Conclusions

Participants responded well to the PHR and they generally found it easy to use. The implementation was feasible and the majority of participants adopted the technology.

Participants did access their test results and imaging reports online and the perceived value of this information was high. However, despite the perceived value of improved understanding and decision making through access to PHI, access itself did not result in improved self-efficacy scores. Having access to personal health information did not increase anxiety levels. Previous studies have not directly measured the impact of access to PHI on participant anxiety levels. Further research is required to investigate how to optimize the delivery of personal health information to patients with different types of cancer, as well as assessing the impact on the patients' experiences, their participation in their care, their relationships with their health care team, and, potentially, their health outcomes.

## Abbreviations

CIHR: Canadian Initiatives for Health Research; EHR: Electronic Health Record; MRN: Medical Record Number; PHI: Personal Health Information; PHR: Personal Health Record; PHWSUQ: Perceived Health Web Site Usability Questionnaire; RTC: Research Triage Centre; SIMS: Shared Information Management Systems; STAI: State Trait Anxiety Inventory; UHN: University Health Network

## Competing interests

The authors declare that they have no competing interests.

## Authors' contributions

DW participated in all aspects of the study and is the lead author on the final manuscript. KL participated in the design of the study and the analysis of the results and contributed to the final manuscript. SU participated in all aspects of the study and contributed to the final manuscript. EA was responsible for data management and contributed to the final manuscript. CM contributed to the data analysis of the final manuscript. NQ was responsible for data management and contributed to the final manuscript. PC participated in the design of the study and contributed to the final manuscript. All authors read and approved the final manuscript.

## Pre-publication history

The pre-publication history for this paper can be accessed here:

http://www.biomedcentral.com/1472-6947/10/46/prepub
